# Vaccine market and production capabilities in the Americas

**DOI:** 10.1186/s40794-021-00135-5

**Published:** 2021-04-13

**Authors:** Esteban Ortiz-Prado, Estefanía Espín, Jorge Vásconez, Nathalia Rodríguez-Burneo, Nikolaos C. Kyriakidis, Andrés López-Cortés

**Affiliations:** 1 One Health Research Group, Universidad de las Américas, Quito, Ecuador Calle de los Colimes y Avenida De los Granados, 170137 Quito, Ecuador; 2 Department of Cell Biology, Physiology and Immunology, Universidad de Barcelona, Barcelona, Spain; 3 Centro de Investigación Genética y Genómica, Facultad de Ciencias de la Salud Eugenio Espejo, Universidad UTE, Quito, Ecuador; 4 Red Latinoamericana de Implementación y Validación de Guías Clínicas Farmacogenómicas (RELIVAF-CYTED), Quito, Ecuador

**Keywords:** Vaccines, Latin America, Market, Vaccine coverages, Economic dependence

## Abstract

In the Americas, The United States of America, Canada, Mexico, and Brazil are the top vaccine producers and the countries with the leading infrastructure for biological manufacturing. The North American countries have the most demanding legislation regulating and controlling these pharmaceuticals’ distribution and production. Some Latin American countries rank in the top 20 of worldwide vaccine manufacturers, with Cuba, Brazil, México and Colombia have a self-sufficient vaccine production of 72.7%, 54,2%; 25%; and 7.7%, respectively, of the national vaccine demand. On the other hand, the rest of Latin American countries cannot satisfy their demand for vaccines, and most of their efforts are associated with the distribution within their health systems rather than in transferring technology.

Based on this literature review, the results suggest an increasing growth vaccine demand, not only for their growing populations and previously established demand but also for the recently exerted pressure due to the COVID-19 pandemic.

Because the American continent has a marked inequality between the hegemonic producers of vaccines, the exporters, and those that depend heavily on importing these products, this could assert technological dependence in countries with rapid population growth and jeopardize the effectiveness of the two vaccination plans.

## Introduction

It is well known that vaccination against various diseases, including preventable, contagious, and life-threatening illnesses, is the best public health intervention after water sanitation. Vaccinations aim to save millions of lives by generating artificial and life-saving immunological responses [1].

**Fig. 1:**
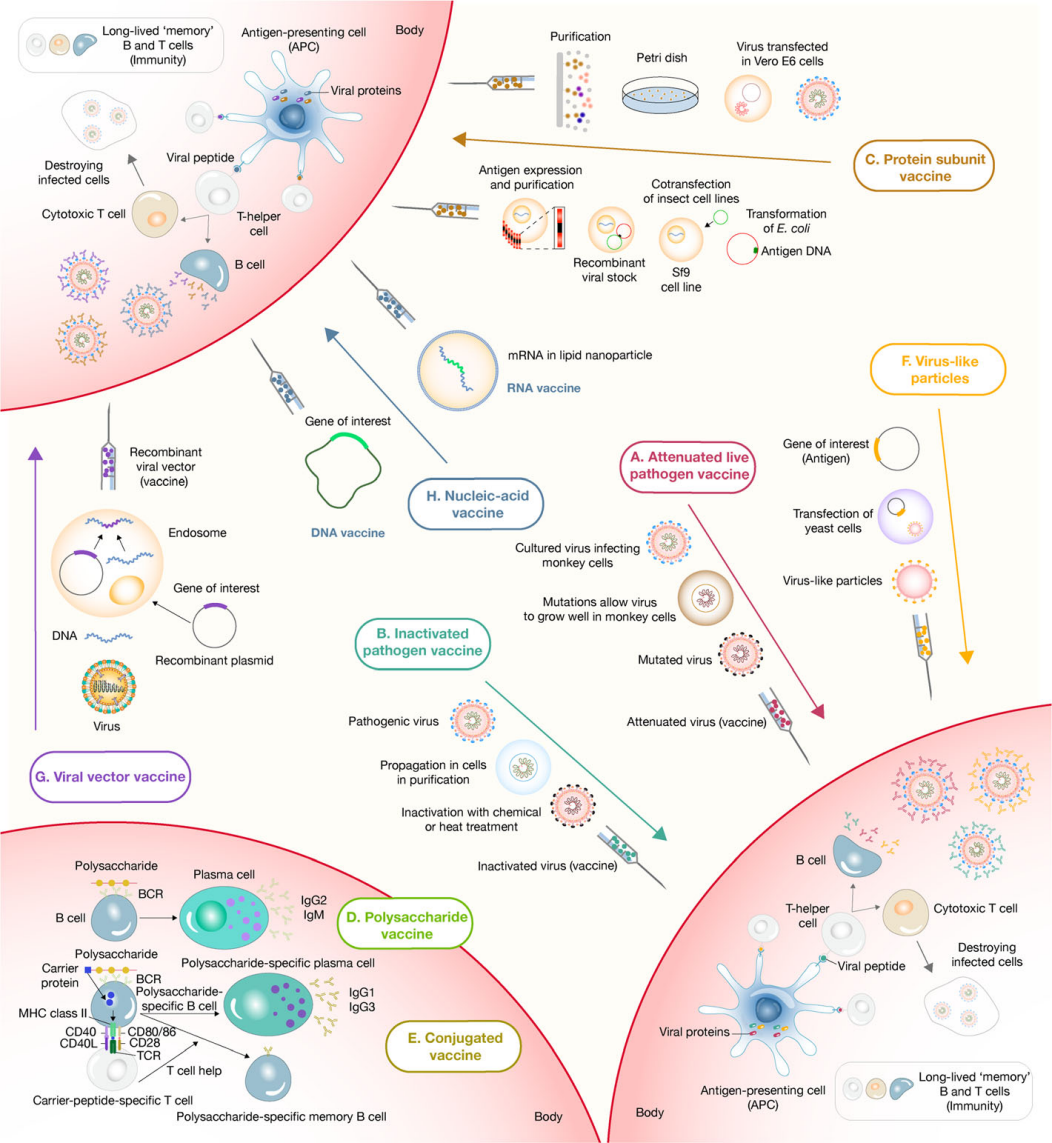
Panoramic landscape of the strategies used for vaccine development and delivery. **a** The live attenuated vaccine strategy consists of administering a weakened form of a live pathogen. This strategy usually elicits robust and long-term memory immune responses after a single dose. **b** The inactivated pathogen vaccine strategy also consists of administering a whole pathogen, but in this case, inactivated by heat or chemical treatment. This strategy has a better safety profile but often is found to be less immunogenic. **c** In contrast with whole pathogen strategies, subunit vaccine strategies include administering only the most immunogenic antigens of a pathogen. These strategies commonly require adjuvant addition to triggering robust immune responses. Protein subunit vaccines are the most prominent members of this group. **d** Another type of subunit vaccine is polysaccharide vaccines. This strategy consists of administering long chains of sugar molecules that make up certain bacteria’s surface capsules. However, polysaccharides are T-independent antigens, eliciting low quality and short-term immune responses, especially in children. **e** To tackle this disadvantage, conjugated vaccines were perceived. This strategy uses a molecule composed of a polysaccharide antigen fused or conjugated to a carrier-protein antigen that induces long-term immunological memory, isotype switching, and affinity maturation processes. **f** The virus-like particle strategy is the last member of the subunit vaccine group. In this case, antigens are expressed in recombinant yeast cells, vaccinia virus or tobacco mosaic virus transfected organisms, and subsequently self-assembled in virus-like particles whose size and conformation induce their capture from host antigen-presenting cells, eliciting solid immune responses post-administration. **g** The viral vector vaccine strategy consists of applying a genetically manipulated measles or adenoviral platform to express a foreign antigen to trigger robust immune responses. **h** Finally, the nucleic acid vaccine strategies (DNA and mRNA) codify the pathogen’s immunogenic proteins. Once administered, the genetic material is captured by myocytes or antigen-presenting cells that use it to express and present the antigen to the host immune system

Over the past few decades, mankind has experienced rapid progress in developing new vaccines, including recent novel coronavirus (COVID-19) vaccine developments that added more than 200 candidates to the production pipeline (Fig. 1) [2].

Around the globe, several production plants manufactured vaccines for decades. Most of these production plants still use immunological strategies that were developed decades ago. During the nineteenth century, scientists from United States, Great Britain, France, and Germany developed strategies for inactivating a whole bacteria and developed inactivated whole-cell vaccines against plague, typhoid, and cholera. Therefore, it is helpful to contemplate the past for having a great deal of forwarding gazing with new potential vaccine production strategies. For instance, the first influenza vaccine using attenuated virus was developed in 1936, while mRNA and viral-vector-based vaccine have been developed in more modern plants faster than ever before, as is the case of the latest COVID-19 vaccines [3–5] (Fig. 1).

Although it appears that many countries have sufficient national vaccine production capacities, this differs significantly from reality. Recently several countries have decreased vaccination rates below the desirable levels, jeopardizing public health strategies to reduce mortality, morbidity, and prevalence of diseases [6].

The history of vaccine production started with Edward Janner’s and Louis Pasteur’s innovation and immunization practices, making the development of vaccination a necessary practice for improving wealth. Nowadays, vaccine development requires specific industries, a robust economic situation, and significant investments [7]. Vaccine production is directed by states with a technological industry large enough to supply each country’s needs. Many of these countries have an extensive potential for vaccine production, which changes the structure of a technology-based economy [8].

Enormous amounts of money have been put into programs that are trying to reduce preventable diseases, especially in developing countries [9]. Although these capital injections have managed to reduce the impacts of comorbid diseases in some of the world’s most remote parts, they have not yet managed to build independent national vaccine production capacities, making these non-self-sustainable interventions a temporary solution.

Vaccine manufacturing industries are driven primarily by developed countries and production-rated economies. Therefore, most developing and low-resource countries need to import the final product and equipment for quality vaccines [10, 11]. On top of this, pharmaceutical plants’ high costs create difficulties for developing countries to manufacture vaccines or compete with existing manufacturers (Table 1).

**Table 1:** analysis of production costs linked to the development of different vaccines [10]

Costs	Cost range
Product development	> 500 M USD
Facilities and Equipment	50 to 700 M USD
Direct Labor	Typically, less than 25% of the total manufacturing cost
Overhead	Up to 45% of the cost of raw materials and labor
Licensing/Regulatory and commercialization	Simple vaccine: - Evaluation fee of 25 to 100 K, and Annual fees of 4.8 K to 140 K USD Combo or Novel Vaccines: - Evaluation fee of 66.5 to 232.8 K USD, and Annual fees of 8.4 to 250 K USD

Although the budget for vaccination programs has increased drastically over the past two decades, some countries still experience low coverages.

In this sense, it is a priority for countries to have suitable and self-sustainable policies as well as enough political will to allocate all the necessary monetary resources to maintain an adequate and constant supply of vaccines and well-established immunization programs to guarantee their distribution in those places [12].

Relocating resources and creating public policy that aims to manufacture vaccines is challenging for everyone, especially for low-and-middle-income countries. The presence of political instability, poor infrastructure, dubious regulatory frameworks, and other factors are some of the barriers to save vaccine production.

This review aims to identify the information available concerning the productive capacities of the American continent’s different countries, including The United States of America and Canada and countries such as Bolivia, Cuba, Peru, and Ecuador.

### Vaccines in the Americas

The World Health Organization (WHO) established the Expanded Program on Immunization (EPI) in 1974 to develop and expand immunization programmes throughout the world; the goal was child’s immunization against tuberculosis, diphtheria, poliomyelitis, pertussis, measles, and tetanus [13]. The WHO Regional Office for the Americas (PAHO) developed an EPI in 1977 and established the Revolving Fund, a solidary and equitable mechanism to facilitate vaccine acquisition to the region’s countries. The first EPI recommended the use of vaccines to protect against six diseases: tuberculosis (BCG), diphtheria, tetanus, pertussis (DTP), measles (Meas), and poliomyelitis (IPV) [14]. Vaccine development between 2000 and 2010 introduced to the EPI: combined vaccines with several antigens Diphtheria whole-cell pertussis tetanus (DwPT) - Hepatitis B (HepB) - Haemophilus influenza type b, inactivated polio vaccine (Hib) – Inactivated Polio vaccine (IPV); pneumococcal conjugate vaccine (PCV); rotavirus (RV), human papillomavirus (HPV), meningococcal (MCV), and yellow fever (YF) vaccines.

The WHO has divided vaccines into three major categories: i) traditional: Diphtheria-Tetanus (DT)-containing, MCVs, DwPT-HepB-Hib, BCG, IPV, HepB, Hepatitis A (HepA); ii) innovator: new vaccines as PCV, RV, meningococcal conjugate vaccine (MCV4), HPV, varicella; and iii) targeted: regional and outbreak (YF). Traditional vaccines lead global market volume, and innovator vaccines drive global market value. Although a few vaccine manufacturers dominate the market (GSK, Sanofi, Serum Institute of India (SII), Microgen and Merck, Central and South American Developing Countries Vaccine Manufacturers (DCVM) supply the highest doses for this region, suggesting a preference for local producers [15].

### Vaccines in Canada

Canada is one of the top producers of vaccines globally, housing several international manufacturers such as GlaxoSmithKline, Merck Canada Ltd., Novartis, Pfizer Canada (formerly Wyeth Pharmaceuticals Canada), and other producers such as Sanofi Pasteur [16]. The vaccine industry in Canada also comprises of national firms such as Solvay Pharma Inc., Medicago, Immunoaccine Inc., Variation Biotechnologies, and many more small biotech companies [17].

Canada has a Vaccine Industry Committee, which works to ensure the supply of vaccines and advocate equitable access as well as regulating quality and safety. This committee brings together some of the largest and most important pharmaceutical companies in the world and government institutions, and civil society [16]. The leading vaccine producers in Canada are Sanofi, which produces and manufactures DTP, BCG, IPV, DT vaccines; and GlaxoSmithKline (GSK), manufactures influenza vaccines. Manufacturers in Canada produce vaccines for both global clinical trials and/or commercial sales [17]. During 2018, vaccine sales were USD 1.822 billion [18]. Despite meeting their national vaccination program requirements with international and national commercial vaccine production and exporting USD 15.208 billion of pharmaceutical products, Canada still imported USD 69.992 billion [19].

Regulation requirements for vaccines in Canada are regulated by Health Canada, which evaluates the safety, efficacy, and quality of a vaccine based on scientific and clinical evidence. Health Canada also authorizes only vaccines that meet this requirement and then monitored the quality and post-market surveillance of vaccines. The Food and Drug Act categorizes vaccines as biological drugs, requiring more regulatory oversights and expertise and procedures for their manufacture, control, and regulation [20].

Vaccination coverage goals in Canada are part of the National Immunization Strategy objectives for 2016–2021. The country has a compulsory vaccination strategy for children of school-age [21]. With the implementation of the “Panorama” system, an integrated public health information system designed to help public health professionals manage vaccine inventories, immunization, investigation, outbreaks, and family health, vaccination is more accessible to the public [22]. As part of the National Immunization Strategy, Canada requires infants to receive pertussis, DTP-Hib, IPV, MMR, chickenpox, and PVC vaccines, and adolescents receive PCV and MCV4 vaccines. HepB is required for all ages [23].

Canada is one of the drivers for the discovery of new vaccines. Recently, the country produced the vaccine that targets cervical cancer caused by HPV and gastroenteritis due to retrovirus. Investment in vaccine development in Canada will lead to vaccines against HIV and malaria [24].

### Vaccines in the United States

The United States of America is the world leader in vaccine production, with two of the largest companies founded 1849 and 1891 by George Merck and Charles Pfizer.

The country’s top vaccine manufacturers are Merck & Co., Inc., which manufactures HPV, HepB, RV, varicella, and varicella-zoster vaccine; Sanofi Pharmaceuticals, which produces Haemophilus b, DTaP, influenza, rabies, IPV, MCV4, TD, and YF vaccines. Johnson & Johnson manufactures experimental Ebola, HIV, RSV, and Zika vaccines. Pfizer produces pneumococcal and MCV4 vaccines [25, 26].

Licensing requests for medical products in the U. S grew substantially between the end of the 1990s and 2010, stimulated by the biotechnology industry’s emergence [27]. In 2019, importations of pharmaceutical products and biological substances were USD 210.1 billion (4.99%) of the total imported products. During the same year, the United States exported USD 59.6 billion (3.26%) of pharmaceutical products. The Pharma industry represented the fastest growing sector among the top ten import and export categories [28].

The institution responsible for regulating vaccines in the US and most world countries is the FDA’s Center for Biologics Evaluation and Research (CBER). They ensure that vaccines are safe and effective. The FDA also oversees the production of vaccines after the vaccine and its manufacturing process is approved. Vaccine clinical development follows the same general pathway as for drugs and other biologics [29].

This unique productive capacity and efficient local regulation, and the incentive for research and development make this country the undisputed leader in vaccines’ world production. The local demands for vaccines are primarily satisfied with local production, and manufacturers allocate a large part of their production for export, creating a significant income for the national economy.

The United States of America recommended vaccines for children and adolescents are HepB, RV, DTaP, Hib, PCV, IPV, MMR, VAR, HepA, Tdap, HPV, MCV4, PVC, and influenza [30]. The US has a program to accelerate vaccines development with an annual budget of $1.00 billion [18]. The most recent numbers stipulate that more than 90% of vaccination coverage is achieved in the country and come mostly from in-country local manufacturers.

In the general context of the COVID-19 pandemic, the US has put the lead on developing several candidates, including two RNA Vaccines, to tackle the effects of COVID-19 in the world. If any of the vaccines reaches the market, the revenue for their economy will be very significant.

### Vaccines in Mexico

The production of vaccines in Mexico began in 1939. In 1970 the WHO recognized the National Institute of Virology as a regional reference center for vaccines. This governmental institution produces vaccines against rabies, MCV, tetanus, and poliomyelitis, covering approximately 90% of vaccines required from 1956 to 1960 [31].

In 1999, the Mexican government founded the firm General Biologic and Reactives from Mexico (BIRMEX). Each year Birmex imports control and distributes about 20 million doses of seasonal and pandemic influenza vaccine delivered to health institutions throughout the country [32]. The company is responsible for manufacturing 25 million OPV doses and 12 million DT doses, which covers the national demand. Birmex plants can produce up to 100 million vaccines a year, as well as two antidotes against the venom of Scorpion and vipers [31].

In 1998, when the measles, mumps, and rubella vaccine (MMR) was introduced, Mexico ceased to be self-sufficient in vaccines production [33]. Therefore, Mexico established a technology transfer agreement with Sanofi Pasteur to produce viral vaccines, like rabies, oral polio vaccine (OPV), and seasonal pandemic influenza vaccines. From 24 vaccines included in its EPI, Mexico ensures 25% of this demand [32].

Currently, the pharmaceutical industry in Mexico is the second largest industry in Latin America and ranks 12th worldwide. The total value of pharmaceutical products is 94 billion Mexican pesos, representing 1.2% of the national gross domestic product [34], and the pharmaceutical sales for anti-infectives for systemic use during 2018 was USD 5.383 billion [18]. During the same year, Mexico imported USD 318 million of vaccines for human use. Meanwhile, exported USD 24.7 million of vaccines primarily to France [34] (Table 2).

**Table 2:** Imports and exports of vaccines for medical use in Mexico during 2018 [34]

Country	Imports	Exports
France	USD 115 million	USD 24.6 million
United Kingdom	USD 56.9 million	No reported
India	USD 33.3 million	No reported
Ireland	USD 29.2 million	No reported
United States	USD 25.9 million	No reported
Belgium-Luxembourg	USD 21 million	No reported
Spain	USD 19.5 million	No reported
Canada	USD 8.37 million	No reported
Switzerland	USD 2.92 million	No reported
No reported Italy	USD 522 thousand	No reported
Germany No reported	USD 508 thousand	No reported
Chinese Taipei	USD 420 thousand	No reported

### Vaccines in Cuba

Over the years, Cuba has developed an interesting capacity to produce health technologies to maintain its national health system. Cuba is recognized for achieving vaccine universal vaccine coverage and other health indicators comparable with high-income countries in the Latin-American context. The biotech sector had its highest recognition in the 1980s, when Cuba had several qualified experts that were trained abroad. After the Soviet Union’s fall, Cuban technological development declined, slowing national production of biotechnological products and biotechnological patents. In any case, this Caribbean country continues to produce research that has allowed it to position itself as the technology transfer leader to South American countries.

The Finlay Institute, the Center of Genetic Engineering and Biotechnology (CIGB), and the National Bio-preparations Centre (BIOCEN) is one of the institutions that leads the production of vaccines. They have improved their technological capacities and produce combined vaccines based on DTwP, such as DTwP-HB and DTwP-HB-Hib, Meningococcal B outer membrane protein vaccines, HepB, HiB, leptospirosis vaccines, polysaccharide Vi Typhoid vaccines, and the meningococcal polysaccharide vaccine ACYW135. In Cuba, there are two vaccine producers: Center for Biotechnology and Bioengineering (CIGB) that produces HepB vaccine, *Haemophilus influenzae* type b vaccine, Tetravalent DPT-HB, Pentavalent DPT-HB-Hib and the Institute of Havana that produces Meningococcal BC vaccine, Trivalent leptospirosis vaccine Vi, polysaccharide typhoid vaccine Tetanus vaccine, DT vaccine, DTwP vaccine [32].

In this sense, Cuba has become self-sufficient in terms of vaccine production in the region, covering more than 72.7% of its national needs with local production.

In recent years, Bio-Manguinhos Institute from Brazil and Cuba have established a cooperation agreement with the Finlay Institute to produce a meningococcal polysaccharide A/C vaccine to eradicate meningitis in Africa. This benefits both countries, including the construction of a good manufacturing procedures (GMP) facility in Cuba for meningococcal polysaccharide production, introducing this vaccine in the Brazilian EPI, WHO prequalification of the product in 2007, and collaborative interaction of their national regulatory agencies [32].

According to data from the national pharmaceutical bureau, the Cuban pharmaceutical industry generated more than 2.7 billion dollars in pharmaceutical products from 2008 to 2013, a remarkable amount considering that Cuban products are exported to many countries in the world [31–33].

### Vaccines in Honduras

Honduras does not produce vaccines for human use. The internal consumption is based on importations and resources that are not produced within the country. The country’s public health needs are part of the EPI program supported by the Panamerican Health Organization (PAHO). The private needs are based on imports by private pharmaceutical companies such as Farsiman, Henie Farma, Kernel, MC Pharmaceutics Corporation, Finlay, Infarma, aiming to prevent infectious diseases that are prevalent such as pneumococcal pneumonia, rotavirus, measles, or mumps [35].

According to latest data, in 2018, Honduras imported vaccines for human use primarily from South Korea (32%), Belgium- Luxembourg (26.8%), India (17.6%), Netherlands (13,3%), Switzerland (4,91%), United States (2.1%), France (2.08%), Indonesia (0.76%), Canada (0.27%), and the U.K (0.09%) [36].

The overall supply of vaccines has contributed to achieved vaccination coverages that are close to 95% in some cases, putting Honduras ahead of other countries in terms of coverage and was recognized by PAHO and WHO for achieving 97% coverage [37]. The primary health services’ mandatory scheme are BCG, Pediatric HepB, OPV, RV, pentavalent (DPT-Hep B- Hib, pneumococcal 13 valent, MRM, RM, DTP, DT, HPV, adult Hepatitis, HepA, influenza, and YF [37].

.Lastly, for some highly needed vaccines and were not available locally, such as the 13-valent pneumococcal vaccine, the government created a joint effort with the Gavi Alliance, PAHO, and UNICEF to supply the amount required by vaccination programs [38].

### Vaccines in Nicaragua

Nicaragua has not produced vaccines; nevertheless, it has near-future plans to build a vaccine production plant that would supply the country and certain countries in the region.

The cooperation among Russia and Nicaragua will result in the construction of a laboratory and manufacturer plant in Managua (ElieMéchnikov), produce vaccines against epidemics, and supply the rest of the Central American countries and the countries of the Bolivarian Alliance for the Peoples of America (ALBA). The research objectives of ElieMéchnikov are the production of vaccines for diseases as dengue, chikungunya, yellow fever, and influenza. It is expected to manufacture at least 30 million flu vaccines per year [39]. ElieMéchnikov laboratory represents an investment of 21 million dollars, of which 7 million were assumed by Nicaragua and Russia assumed 14 million. The laboratory is equipped with technology from the San Petersburg Vaccine and Serum Institute.

According to the Observatory of Economic Complexity (OEC), during 2018, Nicaragua imported USD 9.02 million of human use vaccines, mainly from Belgium-Luxembourg (USD 3.72 million), India (2.17million), France (USD 717 thousand), Russia (USD 602 thousand), The Netherlands (USD 584 thousand), and Turkey (USD 495 thousand) [36]. The Gavi Alliance’s economic contribution to Nicaragua’s Government from 2000 to 2019 is described in Table 3.

**Table 3:** Economic contribution to Nicaragua. Source: Gavi’s Report [40]

Type of support	Approvals 2001–2023 (USD)	Disbursements 2000–2019 (USD)
Health system strengthening (HSS 2)	$3,793,600	$3,793,600
Immunization services support (ISS)	$293,280	$293,280
Injection Safety Devices (NVS)	$74,000	$74,000
Injection safety support (INS)	$461,990	$461,990
Vaccine against Polio	$2,040,500	$2,040,500
Pneumo (NVS)	$16,361,767	$17,915,772
Rotavirus (NVS)	$14,024,262	$12,729,500
Vaccine Introduction Grant (VIG)	$309,935	$309,935
**TOTAL**	**$37,359,334**	**$37,618,577**

### Vaccines in Panama

Panama has one of the best vaccination schemes in the Region of the Americas [41]. Since the creation of the Expanded Immunization Program in 1978, the country has focused its actions on keeping the country free of preventable diseases, making vaccination freely accessible to all citizens [42]. The country’s mandatory vaccines are YF, DT / DTw, MMR, HepA, and HepB vaccines [43]. The leading pharmaceutical supplier is Sanofi [32]. Nonetheless, in 2010 Panama exported large quantities of H1N1 vaccines to Chile and the Central America region [43].

During 2018, Panama imported USD 30.4 million of vaccines for human use, mainly from Belgium-Luxembourg (USD 11.1 million), United States (USD 8.22 million), United Kingdom (USD 3.44 million), France (USD 3.1 million), and Canada (USD 2 million). Meanwhile, exported USD 825 thousand to Costa Rica (USD 634 thousand), Guyana (USD 125 thousand), Ghana (USD 58.7 thousand), and Suriname (USD 5.9 Thousand) [36].

### Vaccines in Colombia

The National Institute of Health in Colombia has an internal capacity to produce BCG vaccine and has improved its laboratories to manufacture a YF vaccine for the international market. Colombia provides 7% of its national immunization needs [32]. In November 2016, a vaccine against malaria was announced, the “Colombian Falciparum Vaccine” (Colfavac). This vaccine was developed by the Foundation Institute of Immunology of Colombia (FIDIC) after 35 years of research [44]. Colfavac is currently ready to be tested in humans after succeeding in pre-clinical trials.

Leading pharmaceutical suppliers of vaccines in Colombia are Biotoscana, Manufacturing Process Prom, Vesalius Pharma, and Sanofi Pasteur. While the primary pharmaceutical producers of vaccines in Colombia are FIDIC, SGS –Vaccines and Biological Products, and Sanofi-Colombia [15].

During 2018, Colombia imported USD 106 million of vaccines for human use, mainly from Belgium- Luxembourg (43.6%), the US (17.5%), France (16.2%), India (8.52%), and South Korea (5.47%) [36].

### Vaccines in Venezuela

In close cooperation with Cuba, Venezuela launched in 2014 a project to build a vaccine production plant. This plant will favor developing local capacities to prepare vaccines against emerging and re-emerging diseases such as tetanus and diphtheria. Since their initial installation, the National Institute of Hygiene Rafael Rangel produces DTwP and rabies vaccine components in their recently built facility.

Although the country’s political and economic deterioration has exacerbated poverty, it appears to be still in operation, administering some locally produced vaccines to the national vaccination scheme. The plant includes large-scale fermentation and purification processes for DTwP antigens equipment, and a facility for the formulation, filling, and packaging of 35 million vaccine doses. According to the information available, the plant can produce 50 million doses once it reaches 100% of its manufacturing capacity.

Although Venezuela imported more than USD 14.2 million of vaccines for human use from India (USD 12.8 million), France (USD 1.27 million), and Denmark (USD 165 thousand) in 2014, the vaccine coverage rates are inconsistent with the official information available [36].

It seems like Venezuela appears to be able to produce DT locally and wP antigens to formulate a pentavalent DTwP-HB-Hib vaccine using a technology transfer model similar to the Cuban System [32]. Although no information regarding safety and efficacy is available, Venezuela reported that a locally produced H1N1 vaccine was used for national vaccination programs [45].

### Vaccines in Peru

Like most Latin American countries, Peru has a limited biotechnology industry and very scarce production of vaccines for human use. In general terms, the Andean country allocates most of its resources to importing vaccines and the local production of a few vaccines for animal use.

In the past, Peru joined the EPI strategy in 1984 with low levels of coverage that did not exceed 20%. Nevertheless, 10 years later, the Andean country achieved an impressive 80% vaccine coverage rate [45, 46]. In 1994, Peru received significant international support to eradicate poliomyelitis and reduced the presence of other infectious diseases that were highly prevalent such as measles and rubella [47]. However, a regression in the country’s immunization efforts fails to reach the minimum standards [48]. Peru is one of the countries within the region, with relatively small production capacities and minimum potential for developing new technologies [32]. However, the Peruvian state funds 100% of the national vaccines program [45, 46].

UNICEF since 1982 has collaborated with the Peruvian immunization program, investing USD 2,200,000 for vaccine acquisition in a campaign against measles and rubella. Additionally, in March 2015, Peru and France launched a cooperative technology transfer project with SANOFI-Pasteur laboratory for vaccine manufacturing [49, 50]. This project’s goals are smallpox, Meas, OPV, rubella, avian influenza, among other viruses and vaccines [49].

During 2018, Peru imported vaccines for human use mainly from Belgium – Luxembourg (USD 27.6 million), United States (USD 16.3 million), France (USD 14.5 million), India (USD 10.6 million), and South Korea (USD 9.76 million), meanwhile exported all its production to Singapore (USD 8.6 thousand) [36].

### Vaccines in Brazil

Brazil is the major vaccine manufacturer in the region. The country is self-sufficient for 54% of vaccination needs, including DTP, DT for adults and infants, TT, HepB, and DTP combined vaccines (DTP-HB and DTP-HB-Hib), seasonal influenza vaccine, YF vaccine, and meningo AþC based on a cooperation agreement between Biomanguinhos and Finlay Institute from Cuba. Other vaccines as MMR, OPV, pneumococcal, and RV vaccines are manufactured due to partnerships and technology transfer agreements with European pharmaceuticals as GlaxoSmithKline (GSK). Butantan Institute is developing other vaccines, such as rabies, RV, and influenza, which will increase regional capacity. In addition, decavalent pneumococcal conjugated vaccine, dengue, and acellular pertussis vaccines are in the scale-up phase, with clinical trials in development [32].

The three leading vaccine producers in Brazil are Butantan Institute San Paolo that elevates DTwP, DT, rabies HB., seasonal, and pandemic influenza vaccine. Fiocruz/Biomanguinhos that produces Tetravalent DPT-Hib, Meningitides A and C polysaccharide, Hib, PCV, YF, OPV, MMR, and RV vaccine; and Technological Institute of Parana was involved in rabies and bacterial vaccine production [32].

The country ensured its vaccine production in Biomanguinhos and Butantan Institute by investing in facilities for the formulation, filling, lyophilizing, final processing, and a technological platform for viral vaccines. In the past, other public laboratories, such as the Institute of Technology of Paraná (TECPAR), were developing bacterial and viral vaccines, but investments in facilities were needed to fulfill GMP [32].

Brazil’s vaccine production is focused on both supplying national needs and for exportation. Vaccines such as for measles and rubella are manufactured for the Bill and Melinda Gates Foundation. This foundation has donated some of these vaccines to developing countries unable to afford them in Africa. Brazil exports mainly triple viral SRP vaccines because these are the most common in the country [51].

Brazil is one of the major worldwide producers of the YF vaccine, with the lowest market price. During 2017 and 2018, Brazil refrained from exporting the vaccine due to the reduction of production. The domestic demand increased significantly due to the need to face an outbreak [52]. The Foundation Oswaldo Cruz, the official vaccine’s producer of the Brazilian Health Ministry, announced to PAHO and UNICEF that from 2019 to 2020, they supply both organizations with 23 million vaccines. WHO certifies these vaccines since 2011 [52].

Human use vaccines in Brazil are imported mainly from Belgium- Luxemburg (USD 273 million), Italy (USD 91.5 million), France (USD 89.9 million), United States (USD44 million), and India (USD 43.2 million). In contrast, the major export destinations are France, Argentina, Colombia, Angola, Chile, and Ecuador [53].

### Vaccines in Bolivia

In 1826, the Medical Institute Sucre (IMS) developed the smallpox vaccine [54]. In 1979, the Bolivarian Government implemented the national immunization program eradicating diseases like poliomyelitis, measles, and rubella. Bolivia imports vaccines mainly from the United States, Belgium- Luxembourg, France, India, and South Korea [38, 52]. Bolivia has acquired vaccines through donations, as well. The national immunization scheme has 11 mandatory vaccines for 17 diseases, including BCG, OPV, Pneumococcal, RV, seasonal antipyretic pediatric, MMR, and DTP vaccines [54].

Bolivia imported all vaccines for national use. During 2018, Bolivia imported USD 17.1 million in vaccines for human use manly from Belgium-Luxembourg (USD 4.62 million), India (USD 3.99 million), South Korea (USD 2.56 million), United States (USD 2.31 million), and France (USD 2.15 million) [36].

### Vaccines in Paraguay

In 2011, Paraguay’s expenses in health and concerning pharmaceuticals were $ 2.985 billion [55]. During 2013 there were 22 pharmaceutical manufacturers in Paraguay, such as LASCA, SA, and Catedral Laboratories SA. There is no robust data about the national production of vaccines. EPI provides free vaccines to children under five, pregnant women, the elderly, and poor people [56].

During 2018, Paraguay exported a total of USD 36.4 thousand in vaccine for human use entirely to Argentina, while it imported USD 12.1 million from the United States (37%), France (22.6%), Belgium-Luxembourg (13%), Canada (9.61%), and India (6.22%) [36].

### Vaccines in Argentina

In Argentina, three different public institutions are involved in the production of vaccines. The Malbrán Institute produces DTP and BCG vaccines in limited quantities not under of GMP. Current goals of production are focused on antiserum for different diseases. The Institute will invest in facilities to manufacture DTwP and combined vaccines based on DTwP. On the other hand, the Institute of Human Viral Diseases Julio Maiztegui built a plant production for vaccines against Argentine hemorrhagic fever that may produce other antiviral vaccines [32, 57]. The live attenuated anti-Argentine hemorrhagic fever vaccine was developed through a joint international effort that envisioned it as an orphan drug [32, 58]. The FDA has not yet approved this vaccine, and it is in the testing phase for approval [59].

Sinergium Biotech is an argentine industry under a technological alliances model, and technological transfer projects develop modern vaccines against influenza, HPV, and pneumococcus. The biotechnological firm started a public-private partnership to expand access to health, supplying vaccines, many of them free-provided, as they integrate the official vaccination schedule [37]. This laboratory is getting ready to distribute 12 million doses of vaccines, with a capacity to supply South America. It is capable of manufacturing the vaccine of zika virus as soon as it is tested and certified. Vaccines are accredited by the National Administration of Medicine, Food and Medical Technology (ANMAT), whose certification is recognized by all South American countries except Brazil [37].

In 2016, an agreement was announced between the company Sinergium Biotech (an Argentine biotechnology company that develops, manufactures, and markets different vaccines, including the flu, for pneumococcus and HPV), the Mundo Sano Foundation, and the North American company Protein Sciences for the development in Argentina of a vaccine against Zika [60].

The vaccine to be developed will be based on the production of recombinant variants of the Zika virus protein E. Other similar vaccines in development produced by Protein Sciences against West Nile virus and Japanese encephalitis, both related to Zika, have demonstrated their ability to neutralize their respective viruses in pre-clinical studies [61].

Argentina imported human vaccines mainly from Italy (USD 47 million), United States (USD 37.4 million), Belgium- Luxemburg (USD 30.7 million), Ireland (USD 20.2 million), and France (USD15.7 million). While the major export destinations are India (USD 818 thousand), Pakistan (USD 534 thousand), Paraguay (USD 317 thousand), and Bolivia (USD 128 thousand) [53].

In Argentina, the National Administration of Laboratories and Institute of Health (ANLIS) “Dr. Carlos G. Malbrán.” Institute of Human Viral Disease“ Julio Maistegui” produces vaccines as Virus Junin live attenuated vaccine against Argentine Hemorrhagic Fever Rabies vaccine (Table 4) [32].

**Table 4:** Novel vaccines produced in Argentina

Institute that provides/ developed	Disease	Description
CONICET, CIDE	Giardiasis	An oral vaccine against giardiasis. According to the WHO, this disease infects 200 million people in Africa, Asia, and Latin America, with a particular impact on children up to 5 years because they are in a development stage where proper nutrition is vital. The purpose of the vaccine is to fight this intestinal parasite, which can lead to eating disorders, severe inadequate absorption syndromes, and whose most severe forms are malnutrition, dehydration, and diarrhea, which in some cases can cause death [62].
IIBBA COMICET, Instituto Leloir	HPV	A pre-clinical staged technology to produce a vaccine against HPV. The technology developed by the researchers consists of assembling a virus identical to the pathogen but “empty.” To create this pseudovirus that activates the system’s immune response, the virus’s capsid is isolated [63, 64].
The Institute for the Study of humoral immunity (IDEHU) and the Institute for Research in medical microbiology and parasitology (IMPAM)	Chagas Disease	Experimental vaccines with preliminary results are promising. The finding refers to studying the effects of a designed molecule that combines the essential immunogenic characteristics of three parasites antigens that causes the disease [65].
Sinergium Biotech	Zika fever	The vaccine is made up of a purified protein called “protein E” that matches a protein naturally found on the surface of the Zika virus. According to early results from pre-clinical studies with mice, the purified protein formulated in the laboratory elicits strong levels of neutralizing antibodies, which is a positive indicator that the vaccine will protect against Zika virus infection [66].

### Vaccines in Uruguay

Uruguay is one of the leading cattle producers of South America; consequently, they have developed research and vaccine manufacturing for cattle, which could be a basis for human vaccine production. The Pasteur Institute of Montevideo and the Clausen Laboratory work together with the Ministry of Public Health for the national production of vaccines [67].

Vaccines are produced to combat seasonal influenza. Authorities recognized that the H1N1 vaccine’s approval could open lines of research involving biochemists, biologists, chemical engineers, and all professionals working in the production of vaccines and the prevention of diseases [68]. This initiative fulfills one of the objectives of the Pasteur Institute in Montevideo. On the other hand, the Clausen Laboratory installed in Uruguay already works with biological products [67]. This small country in South America exports high value-added goods to other countries in the region, including animal vaccines and very sporadic, human-designed biological products (Table 5).

**Table 5:** Exportations of the Uruguayan Pharma sector [61]

Product description	Part (%)
Medications conditioned for retail sale	59%
Vaccines and antiserum	26%
Pharmaceutical preparations and devices	8%
Provitamins and vitamins natural or reproduced by synthesis	3%
Natural or reproduced hormones by synthesis	3%
Other	1%

Uruguay imports human vaccines mainly from the United States (USD 2.46 million), France (USD 2.22 million), Netherlands (USD 2.19 million), South Korea (USD 1.66 million), and Belgium- Luxembourg (USD 1.32 million). At the same time, the major export destinations are Guyana (USD 31.2 thousand), Pakistan (USD 13.7 thousand), and India (USD 3.04 thousand) [53].

### Vaccines in Ecuador

This South American country achieved some historical milestones of biotechnological development, especially with the contributions of the development of vaccines and toxoids such as BCG and the Tetanus vaccine. Ecuador was one of the first countries to produce vaccines in the region. In 1938 the first clinical trials for the BCG vaccine production were carried out, and the vaccine was distributed soon after [69, 70]. Since that beginning, the country has been progressively producing more vaccines, including toxoids and antivenom for human use (Table 6).

**Table 6:** Amounts necessary for the acquisition of vaccines by the PAI in Ecuador and percentage of Imports of vaccines in relation to local production, Source PAI, prepared by the Author [70]

Vaccine name	Revolving fund via EPI	Enfarma / Inspi	% of imports 2013	% Imports 2016
BCG	$ 89.647		100%	100%
MMR	$ 852.194		100%	100%
MR	$ 105.344		100%	100%
Polio Vaccine	$ 402.780		100%	100%
Rotavirus Vaccine	$ 4.011.333		100%	100%
Pneumococcal vaccine	$ 819.275		100%	100%
Immunoglobulin HB.	$ 8.807		100%	100%
Chickenpox vaccine	$ 5.129.735		100%	100%
Yellow fever vaccine	$ 1.585.111		100%	100%
Influenza	$ 6.644.748		100%	100%
Trivalent influenza	$ 3.743.625		100%	100%
Pediatric trivalent influenza	$ 1.032.000		100%	100%
Conjugated Pneumococcal vaccine	$ 15.272.250		100%	100%
DT adults		$ 247.200	39%	100%
Anti-meningococcal		$ 29.960	100%	100%
Hep B		$ 292.500	100%	100%
Pediatric Hep B		$ 25.500	100%	100%
Pentavalent		$ 3.431.000	100%	100%
DPT		$ 92.000	0%	100%
DT Pediatric		$ 72.000	0%	100%
Total	**$ 39.696.850**	**$ 4.190.160**	**88%**	**100%**

After producing vaccines for many years, the only local plant that produced biologicals was forced to close due to the lack of political will and monetary resources [70]. Currently, Ecuador has 18 specific vaccines within the national scheme to prevent infectious diseases, including chickenpox, rubella, pneumococci, HPV, and influenza; nevertheless, all of them are now imported [69, 71].

The latest numbers suggest that Ecuador imports human vaccines mainly from Belgium- Luxembourg (USD 17.5 million), South Korea (USD 10.9 million), India (USD 3.74 million), Russia (USD 3.68 million), France (USD 3.68 million), and France (USD 2.36 million). While the only export destination registered is Panamá [53].

Ecuador is currently facing a reduction in its vaccination coverages according to the Pan-American Health Organization (PAHO) [72]. This reduction might be linked to the disappearance of the local production capabilities, jeopardizing children’s coverages and increasing the risk of future outbreaks.

### Vaccines in other countries from south or Central America

Vaccine production in other smaller countries in the Caribbean, Central or South America is limited. Countries like French Guiana, El Salvador, or Belize have extremely small markets, and the production of biological medicines is not profitable for the target population. Countries like Costa Rica or Puerto Rico have an important and growing pharmaceutical industry; however, the technology to produce their own vaccine is not implemented at a big scale level. Their market’s needs are entirely turned over to the production of generic drugs and branded products sponsored by multinational companies that focus on high-demand products [70, 73].

Finally, some of the not included countries were scarce, not clear, or came from unofficial sources, therefore not discussed in this review; nevertheless, data from most of the region’s countries are displayed in Table 7.

**Table 7:** List of countries in the Americas and their vaccine production capabilities as well as their share in the exportation market

Country	Number of vaccines introduced in the EPI [74]	Vaccine produced in own facility	Local vaccine production^a^ (%)	Ranking in world exports of vaccines for human use [75]	^b^Vaccine Type and target achieved [76]	
Antigua and Barbuda	16	0	0	No information reported	HepB3 Hib3 IPV Pol3 DTP3 MCV2 MCV1 RCV1	99% 99% 99% 95% 95% 95% 93% 93%
Argentina	26	9	34.62	49	MCV1 RCV1 BCG IPV MCV2 PCV3 HepB3 Hib3 DTP3 Pol3 RV YFV	94% 94% 93% 90% 89% 88% 86% 86% 86% 84% 72% 8%
Bahamas	22	0	0	No information reported	IPV Hep3 Hib3 PCV3 Pol3 DTP3 MCV1 RCV1 MCV2 RV	91% 86% 86% 86% 86% 86% 86% 85% 82% 78%
Barbados	15	0	0	63	IPV PCV3 MCV1 RCV1 Pol3 HepB3 Hib3 DTP3 MCV	94% 93% 92% 92% 91% 90% 90% 90% 77%
Belize	16	0	0	No information reported	IPV Hib3 Pol3 DTP3 MCV1 RCV1 BCG MCV2 HepB3	99% 98% 98% 98% 96% 96% 95% 95% 98%
Bolivia	14	1	7.14	No information reported	IPV BCG MCV1 RCV1 RV YFV HepB3 Hib3 PCV3 Pol3 DTP MCV2	81% 80% 79% 78% 77% 75% 75% 75% 75% 75% 75% 44%
Brazil	27	13	48.15	32	MCV1 RCV1 IPV Poli3 PCV3 RV HepB3 Hib3 BCG DTP3 YFV MCV2	91% 91% 86% 85% 84% 83% 80% 80% 79% 73% 60% 54%
Canada	21	5	23.81	11	IPV Hib3 Pol3 DTP3 MCV1 RCV1 MCV2 PCV3 RV HepB3	94% 91% 91% 91% 90% 90% 87% 81% 79% 74%
Chile	19	0	0	No information reported	IPV BCG HepB3 Hib3 Pol3 DTP3 MCV1 PCV3 RCV1 MCV2	99% 98% 96% 96% 96% 96% 95% 95% 95% 91%
Colombia	22	3	13.64	54	MCV1 RCV1 PCV3 IPV HepB3 Hib3 Pol3 DTP3 RV BCG MCV2 YFV	95% 95% 94% 93% 92% 92% 92% 92% 90% 89% 88% 87%
Costa Rica	15	0	0	74	HepB3 IPV MCV1 PCV3 RCV1 DTP3 Hib3 Pol3 MCV2 BCG RV	98% 96% 95% 95% 95% 95% 94% 94% 93% 88% 59%
Cuba	16	9	56.25%	43	BCG HepB3 Hib3 IPV MCV1 Pol3 RCV1 DTP3 MCV2	99% 99% 99% 99% 99% 99% 99% 99% 99%
Dominica	14	0	0	No information reported	HepB3 Hib3 IPV Pol3 DTP BCG MCV1 RCV1 MCV2	99% 99% 99% 99% 99% 98% 92% 92% 92%
Dominican Republic	18	0	0	No information reported	BCG IPV MCV1 RCV1 Pol3 DTP3 HepB3 RV Hib3 PCV3 MCV2	99% 98% 96% 96% 92% 89% 87% 80% 79% 70% 60%
Ecuador	18	2	11.1	No information reported	BCG HepB3 Hib3 IPV Pol3 RV DTP3 YFV MCV1 PCV3 RCV1 MCV2	86% 85% 85% 85% 85% 85% 85% 84% 83% 83% 83% 76%
El Salvador	19	0	0	No information reported	IPV Hib3 Pol3 DTP3 MCV1 RCV1 MCV2 BCG HepB3 PCV3 RV	81% 81% 81% 81% 82% 82% 87% 78% 81% 82% 82%
Grenada	14	0	0	No information reported	IPV HepB3 Hib3 MCV1 Pol3 RCV1 DTP3 MCV2	96% 94% 94% 94% 94% 94% 92% 82%
Guatemala	18	0	0	No information reported	IPV Hib3 Pol3 DTP3 MCV1 RCV1 MCV2 PCV3 RV HepB3	90% 86% 79% 85% 90% 90% 78% 88% 86% 86%
Guyana	16	0	0	81	BCG HepB3 Hib3 IPV RV DTP3 MCV1 PCV3 RCV1 Pol3 YFV MCV2	99% 99% 99% 99% 99% 99% 98% 98% 98% 97% 94% 92%
Haiti	9	0	0	No information reported	IPV Pol3 BCG MCV1 RCV1 HepB3 Hib3 DTP3 RV PVC3 MCV2	78% 74% 73% 65% 65% 51% 51% 51% 48% 42% 41%
Honduras	20	0	0	No information reported	IPV BCG RV HepB3 Hib3 MCV1 PCV3 Pol3 RCV1 DTP3 MCV2	90% 88% 88% 87% 87% 87% 87% 87% 87% 87% 85%
Jamaica	16	0	0	No information reported	BCG IPV HepB3 Pol3 DTP3 Mcv1 RCV1 MCV2	97% 97% 96% 96% 96% 94% 94% 92%
Mexico	20	7	35	35	IPV Hib3 Pol3 DTP3 MCV1 RCV1 MCV2 PCV3 RV HepB3	84% 82% 82% 82% 73% 73% 73% 86% 82% 56%
Nicaragua	15	4	26.67	31	IPV MCV1 Pol3 RCV1 MCV2 BCG HepB3 Hib3 PCV3 RV DTP3	99% 99% 99% 99% 99% 98% 98% 98% 98% 98% 98%
Panama	25	1	4	34	BCG MCV1 RCV1 MCV2 IPV PCV3 RV HepB3 Hib3 Pol3 DTP3 YFV	99% 97% 97% 97% 96% 96% 94% 88% 88% 88% 88% 7%
Paraguay	26	0	0	No information reported	YFV PCV3 IPV BCG MCV1 RCV1 HepB3 Hib3 RV DTP Pol3 MCV2	92% 89% 88% 87% 87% 87% 86% 86% 86% 86% 84% 83%
Peru	20	0	0	No information reported	RV IPV HepB3 Hib3 DTP Pol3 MCV1 RCV1 BCG PCV3 MCV2 YFV	90% 89% 88% 88% 88% 87% 85% 85% 81% 80% 66% 57%
Saint Kitts and Nevis	13	0	0	No information reported	BCG MCV2 HepB3 Hib3 IPV MCV1 RCV1 Pol3 DTP3	99% 98% 97% 97% 97% 97% 97% 96% 96%
Saint Vincent and the Grenadines	13	0	0	No information reported	BCG MCV1 Pol3 RCV1 MCV2 HepB3 Hib3 DTP3 IPV	99% 99% 99% 99% 99% 97% 97% 97% 96%
Suriname	17	0	0	No information reported	IPV HepB3 Hib3 DTP3 Pol3 MCV1 RCV1 MCV2 YFV	82% 77% 77% 77% 76% 64% 64% 58% 57%
Trinidad and Tobago	16	0	0	83	MCV1 RCV1 YFV HepB3 Hib3 PCV3 Pol3 DTP3 IPV MCV2	99% 99% 98% 93% 93% 93% 93% 93% 92% 92%
United States	30	18	60	5	IPV Hib3 Pol3 DTP3 MCV1 RCV1 MCV2 PCV3 RV HepB3	97% 91% 93% 94% 90% 90% 95% 92% 74% 91%
Uruguay	19	2	10.53	No information reported	BCG IPV MCV2 MCV1 RCV1 PCV3 HepB3 Hib3 DTP3 Pol3	99% 99% 99% 96% 96% 95% 94% 94% 94% 93%
Venezuela	16	4	25	No information reported	MCV1 RCV1 BCG YFV HepB3 Hib3 DTP3 Poli3 IPV MCV2	93% 93% 91% 80% 64% 64% 64% 62% 55% 13%

^a^The percentage of self-sufficiency in vaccine production is estimated as the number of different types of vaccines produced by national manufacturers as a function of the number of vaccines in use by the immunization program

^b^*BCG* bacille Calmette–Guerin, *HepB* hepatitis B virus, *DTP* diphtheria, tetanus, and pertussis, *MMR* mumps, measles, and rubella, *Hib Haemophilus influenzae* type B, *HPV* human papillomavirus, *MCV vaccine* meningococcal conjugate vaccine, *DPT* diphtheria-tetanus-pertussis

## Discussion

Vaccine manufacturing is a universal and essential activity to tackle some of the main public health problems worldwide. EPI has been successfully introduced and improved in the American continent, reducing the morbidity and mortality of essential diseases in the region. However, vaccines production has concentrated in developed countries from North America, such as Canada, the United States, and developing countries in Latin America. Cuba, Brazil, México, and Colombia have a self-sufficient production of 72.7, 54.2%; 25%; and 7.7% of the national vaccine demand, respectively [32]. On the other hand, the rest of Latin American countries cannot produce vaccines or vaccine components, depending on external production to satisfy their national immunization programs. The strategies of countries who maintain public manufacturing of vaccines in Latin America include investment in technology and science, improvement of installed capacities to fulfill GMP, and developing industrial capacities with anticipated policies, in concordance with the country’s needs. Besides, collaborative agreements among national institutes and different countries have demonstrated an effective strategy in vaccine manufacturing. Technology transfer programs with the private pharmaceutical industry, as the mechanisms for transferring technology and technical support, guarantee countries’ capacities. Currently, Latin American countries represent a crucial vaccine market in value and volume.

Despite some of the issues in terms of resources, infrastructure, and economic investment, it is also important to emphasize that some countries are in the race to produce vaccines, especially those aimed at controlling neglected tropical diseases [77].

Although the vast majority of clinical studies on vaccines take part in North American countries such as Canada or the United States, some Latin American countries have made progress in carrying out some clinical studies to verify their vaccines’ efficacy and safety (Table 8).

**Table 8:** Information about country-specific vaccines, subtype, current production/clinical trials status, comments about limitations and other limitations

Country	Type of vaccine	Leading Institute	Immunological strategy	Stage of development	Comments/limitations and possible side effects
Argentina	Junin virus (JUNV) vaccine against Argentinian hemorrhagic fever	National Institute of Human Viral Diseases (ANLIS), Argentina	Cell culture with the virus molecular constructs [78].	Based on phase 3, Clinical Trial: Candid#1 vaccine against Argentine hemorrhagic fever produced in Argentina is authorized for marketing in Argentina. Immunogenicity and safety [79] No Clinical Trial Registry. FDA’s Investigational New Drug Application #2257	With the use of the vaccine in high-risk individuals, AHF incidence has declined, but cases continue to be reported [80].
Brazil	Tetravalent Dengue Vaccine	Butantan and the National Institutes of Health (NIH)	Live attenuated tetravalent DEN virus to provide immunity to each of the four serotypes of DEN [81]	NCT02406729, Phase 3, Ongoing. NCT01696422, Phase 2 results demonstrated safety and induced robust, balanced, neutralizing antibody responses against the four DENV serotypes, in both naive and pre-exposed volunteers, after a single dose [82].	Limitation of the previous unique vaccine approved CYD-DTV has shown that age and previous exposure status of vaccine recipients to dengue virus had a significant effect on the safety and efficacy of the vaccine [82]. The tetravalent vaccine expects to overcome this limitation.
	Zika Vaccine	Butantan Institute	Live, DENV-vectored vaccine expressing pre membrane/ membrane and envelope proteins Purified inactivated virus	Early-stage research [83]	
		Bio-Manguinhos	Purified inactivated virus YF17DD chimera VLP DNA		
Colombia	Malaria SPf66 vaccine	Malaria Vaccine and Drug Development Center, Colombia	Synthetic vaccine against the asexual blood stages of *Plasmodium falciparum*	No registry in Clinicaltrials.gov Results published concluded that synthetic SPf66 vaccine may be used as a safe and highly immunogenic vaccine, suitable to protect high-risk populations such as children under 5 years of age resident in hyper- and holo-endemic areas, such as Africa or some regions of Latin America [84]	A posterior Cochrane systematic review reported no evidence for protection by SPf66 vaccines against *P. falciparum* in Africa and a modest reduction in attacks of *P. falciparum* in South America. Further research with SPf66 vaccines in South America or with new formulations of SPf66 may be justified [85]
	Malaria vaccine against *Plasmodium vivax*	Malaria Vaccine and Drug Development Center, Colombia	Vaccine derived from the synthetic CS protein of *Plasmodium vivax*	NCT02083068, Phase II, completed, no results posted	
Mexico	Vacuna Antipoliomielítica Bivalente Oral	Birmex		NCT01870206 Phase 3 Randomized Clinical Trial to Evaluate Immunogenicity and Safety in Mexican Newborns. Status Unknown	

All local vaccine production strategies appear to be directly related to the best vaccination coverage in their countries. Some countries that have stopped producing vaccines have lowered their coverage levels, as shown by the Ecuadorian experience [86].

In contrast to Ecuador, which dropped coverage when manufacturing decreased, Nicaragua, which imports all its vaccines, has exceptionally high vaccination rates, suggesting that manufacturing is not as crucial as a well-funded vaccination program with robust public health and political backing.

On the other hand, it is essential to understand that vaccines are not produced in most countries of the continent, and only those with stronger economies or emerging economies have managed to maintain and generate sustainable and effective production levels.

When we review the region’s current situation, we realize that the countries with the most significant investment in research and development have installed capacities to produce these products with high added value. It is essential to emphasize the need for countries with little or no vaccine production to invest in personnel and to improve technological capabilities for vaccine production. This biotechnological development area will serve shortly to break out of economic dependence and shift to an economy that exports valuable raw materials at the international level.

## Conclusions

This review has demonstrated the marked difference in the productive capacities of vaccines at the continental level. In the American continent, countries with great economies and adequate management of research resources such as Mexico, Canada, or the United States are the leaders in producing and exporting vaccines. In contrast, other countries with emerging economies such as Brazil, Mexico, or Argentina have local capacities installed, and they produce the much-needed vaccines to satisfy their needs and, in some circumstances, regional needs. On the other hand, many developing countries of the region do not start with their bio-industrialization and have little manufacturing capacities or few strategies to become independent in biotech dependence. However, most of the research carried out in these countries has not yet reached a level of industrial scale-up and is dependent on financing and investment by private companies.

Finally, we observe that lower or scarce vaccine production is associated with poorer vaccine rate coverages. Although many other factors are related to this situation, producing biological products in situ will improve capacities and responses, especially in new and emerging diseases.

## Data Availability

Most of the data publicly available, nevertheless, very few databases are available, and the information is dispersed.

## Abbreviations

ANLIS: National Administration of Laboratories and Institute of Health
ANMAT: National Administration of Medicine, Food and Medical Technology
BCG: Bacillus Calmette-Guérin
BIOCEN: National Bio-preparations Centre
BIRMEX: General Biologic and Reactives from Mexico
CETA: Comprehensive Economic and Trade Agreement
CIGB: Center of Genetic Engineering and Biotechnology
DCVM: Developing Countries Vaccines Manufactures
DT: Diphteria-Tetanus
DTG: Diphteria-Tetanus-Pertussis
DwPT: Diphtheria whole-cell pertussis tetanus
EPI: Expanded Program on Immunization
FIDIC: Foundation Institute of Immunology of Colombia
GMP: Good manufacturing procedures
GSK: GlaxoSmithKline
HepA: Hepatitis A
HepB: Hepatitis B
Hib-IPV: Haemophilus influenza type b, inactivated polio vaccine
HPV: Human papilloma virus
IMS: Medical Institute Sucre
IPV: Inactivated Polio vaccine
MCV: Meningococcal vaccie
MCV1: Measles-containing- vaccine- first- dose
MCV2: Measles-containing-vaccine second- dose
MCV4: Meningococcal conjugate vaccine
Meas: Measles-only vaccine
MMR: Measles, mumps and rubella vaccine
OEC: Observatory of Economic Complexity
ONEI: National Statistics and Information Office of Cuba
OVP: Oral polio vaccine
PAHO: Pan American Health Organization
PVC: Pneumococcal conjugate vaccine
RV: Rotavirus
SII: Serum Institute of India
TECPAR: Institute of Technology of Panamá
VLP: Virus-like particle
VSV: Vesicular stomatitis virus
WHO: World Health Organization
YF: Yellow Fever
